# Evaluating the Effectiveness of Personalized Medicine With Software

**DOI:** 10.3389/fdata.2021.572532

**Published:** 2021-05-18

**Authors:** Adam Kapelner, Justin Bleich, Alina Levine, Zachary D. Cohen, Robert J. DeRubeis, Richard Berk

**Affiliations:** ^1^ Department of Mathematics, Queens College, CUNY, Queens, NY, United States; ^2^ Department of Statistics, The Wharton School of the University of Pennsylvania, Philadelphia, PA, United States; ^3^ Department of Psychology, University of Pennsylvania, Philadelphia, PA, United States

**Keywords:** personalized medicine, inference, bootstrap, treatment regimes, randomized comparative trial, statistical software

## Abstract

We present methodological advances in understanding the effectiveness of personalized medicine models and supply easy-to-use open-source software. Personalized medicine involves the systematic use of individual patient characteristics to determine which treatment option is most likely to result in a better average outcome for the patient. Why is personalized medicine not done more in practice? One of many reasons is because practitioners do not have any easy way to holistically evaluate whether their personalization procedure does better than the standard of care, termed *improvement*. Our software, “Personalized Treatment Evaluator” (the R package PTE), provides inference for improvement out-of-sample in many clinical scenarios. We also extend current methodology by allowing evaluation of improvement in the case where the endpoint is binary or survival. In the software, the practitioner inputs 1) data from a single-stage randomized trial with one continuous, incidence or survival endpoint and 2) an educated guess of a functional form of a model for the endpoint constructed from domain knowledge. The bootstrap is then employed on data unseen during model fitting to provide confidence intervals for the improvement for the average future patient (assuming future patients are similar to the patients in the trial). One may also test against a null scenario where the hypothesized personalization are not more useful than a standard of care. We demonstrate our method’s promise on simulated data as well as on data from a randomized comparative trial investigating two treatments for depression.

## 1 Introduction

Personalized medicine, sometimes called “precision medicine” or “stratified medicine” (Smith, 2012), is a medical paradigm offering the possibility for improving the health of individuals by judiciously treating individuals based on his or her heterogeneous prognostic or genomic information (Zhao and Zeng, 2013). These approaches have been described under the umbrella of “P4 medicine,” a systems approach that combines predictive, personalized, preventive and participatory features to improve patient outcomes (Weston and Hood, 2004; Hood and Friend, 2011). And the interest in such personalization is exploding.

Fundamentally, personalized medicine is a statistical problem. However, much recent statistical research has focused on how to best estimate *dynamic treatment regimes* or *adaptive interventions* (Collins et al., 2004; Chakraborty and Murphy, 2014). These are essentially strategies that vary treatments administered over time as more is learned about how particular patients respond to one or more interventions. Elaborate models are often proposed that purport to estimate optimal dynamic treatment regimes from multi-stage experiments (Murphy, 2005b) as well as the more difficult situation of inference in observational studies.

Thus, the extant work, at least in the field of statistics, is highly theoretical. There is a dearth of software that can answer two fundamental questions practitioners will need answered before they can personalize future patients’ treatments: 1) How much better is this personalization model expected to perform when compared to my previous “naive” strategy for allocating treatments? 2) How confident can I be in this estimate? Can I reject a null hypothesis that it will perform no better than the standard of care? Chakraborty and Moodie (2013), page 168 believe that “more targeted research is warranted” on these important questions; and the goal of our paper is to provide a framework and usable software that fills in part of this gap.

Personalized medicine is a broad paradigm encompassing many real-world situations. The setting we focus on herein is a common one: using previous randomized comparative/controlled trial (RCT) data to be able to make better decisions for future patients. We consider RCTs with two treatment options (two-arm), with one endpoint measure (also called the “outcome” or “response” which can be continuous, binary or survival) and where the researchers also collected a variety of patient characteristics to be used for personalization. The practitioner also has an idea of a model of the response (usually a simple first-order interaction model). Although this setting is simplistic, our software can then answer the two critical questions listed above.

Our advances are modest but important for practitioners. 1) Practitioners now have easy-to-use software that automates the testing of their personalization models. 2) We introduce a more intuitive metric that gauges how well the personalization is performing: “improvement” vs. a baseline strategy. 3) Our estimates and hypothesis tests of improvement are all cross-validated, making the estimates honest even when the data at hand was overfit and thereby generalizable to future patients. This external validity is only possible if future patients can be thought to come from the same population as the clinical trial, a generalization that is debated but beyond the scope of our work. 4) We have extended this well-established methodology to the setting of binary and survival endpoints, the most common endpoints in clinical trials.

The paper proceeds as follows. In Section 2, we review the modern personalized medicine literature and locate our method and its limitations within. Section 3 describes our methods and its limitations in depth, by describing the conceptual framework emphasizing our methodological advances. We then carefully specify the data and model inputs, define the improvement metric, and illustrate a strategy for providing practitioners with estimates and inference. Section 4 applies our methods to 1) a simple simulated dataset in which the response model is known, 2) a more complicated dataset characterized by an unknown response model and 3) a real data set from a published clinical trial investigating two treatments for major depressive disorder. Section 5 demonstrates the software for all three types of endpoints: continuous, binary and survival. Section 6 concludes and offers future directions of which there are many.

## 2 Background

Consider an individual seeking one of two treatments, neither of which is known to be superior for all individuals. “What treatment, by whom, is most effective for this individual with that specific problem, and under which set of circumstances?” (Paul, 1967).^1^ Sometimes practitioners will select a treatment based informally on personal experience. Other times, practitioners may choose the treatment that their clinic or peers recommend. If the practitioner happens to be current on the research literature and there happens to be a published RCT whose results have clear clinical implications, the study’s superior treatment (on average) may be chosen.

Each of these approaches can sometimes lead to improved outcomes, but each also can be badly flawed. For example, in a variety of clinical settings, “craft lore” has been demonstrated to perform poorly, especially when compared to very simple statistical models (Dawes, 1979). It follows that each of these “business-as-usual” *treatment allocation procedures* can in principle be improved if there are patient characteristics available which are related to how well an intervention performs.

Personalized medicine via the use of patient characteristics is by no means a novel idea. As noted as early as 1865, “the response of the average patient to therapy is not necessarily the response of the patient being treated” (translated by Bernard, 1957). There is now a substantial literature addressing numerous aspects of personalized medicine. The field is quite fragmented: there is literature on treatment-covariate interactions, locating subgroups of patients as well as literature on personalized treatment effects estimation. However, a focus on inference is rare in the literature and available software for inference is negligible.

Byar (1985) provides an early review of work involving treatment-covariate interactions. Byar and Corle (1977) investigates tests for treatment-covariate interactions in survival models and discusses methods for treatment recommendations based on covariate patterns. Shuster and van Eys (1983) considers two treatments and proposes a linear model composed of a treatment effect, a prognostic factor, and their interaction. Using this model, the authors create confidence intervals to determine for which values of the prognostic factor one of two treatments is superior.

Many researchers also became interested in discovering “qualitative interactions,” which are interactions that create a subset of patients for which one treatment is superior and another subset for which the alternative treatment is superior. Gail and Simon (1985) develop a likelihood ratio test for qualitative interactions which was further extended by Pan and Wolfe (1997) and Silvapulle (2001). For more information and an alternative approach, see Foster (2013).

Much of the early work in detecting these interactions required a prior specification of subgroups. This can present significant difficulties in the presence of high dimensionality or complicated associations. More recent approaches such as Su et al. (2009) and Dusseldorp and Van Mechelen (2014) favor recursive partitioning trees that discover important nonlinearities and interactions. Dusseldorp et al. (2016) introduce an R package called QUINT that outputs binary trees that separate participants into subgroups. Shen and Cai (2016) propose a kernel machine score test to identify interactions and the test has more power than the classic Wald test when the predictor effects are non-linear and when there is a large number of predictors. Berger et al. (2014) discuss a method for creating prior subgroup probabilities and provides a Bayesian method for uncovering interactions and identifying subgroups. Berk et al. (2020) uses trees to both locate subgroups and to provides valid inference on the local heterogeneous treatment effects. They do so by exhaustively enumerating every tree and every node, running every possible test and then providing valid post-selection inference (Berk et al., 2013b).

In our method, we make use of RCT data. Thus, it is important to remember that “clinical trials are typically not powered to examine subgroup effects or interaction effects, which are closely related to personalization … even if an optimal personalized medicine rule can provide substantial gains, it may be difficult to estimate this rule with few subjects” (Rubin and van der Laan, 2012). This is why a major bulk of the literature does not focus on finding covariate-treatment interactions or locating subgroups of individuals, but instead on the entire model itself (called the “regime”) and that entire model is then used to sort individuals. Holistic statements can then be made on the basis of this entire sorting procedure. We turn to some of this literature now.

Zhang et al. (2012a) consider the context of treatment regime estimation in the presence of model misspecification when there is a single-point treatment decision. By applying a doubly robust augmented inverse probability weighted estimator that under the right circumstances can adjust for confounding and by considering a restricted set of policies, their approach can help protect against misspecification of either the propensity score model or the regression model for patient outcome. Brinkley et al. (2010) develop a regression-based framework of a dichotomous response for personalized treatment regimes within the rubric of “attributable risk.” They propose developing optimal treatment regimes that minimize the probability of a poor outcome, and then consider the positive consequences, or “attributable benefit,” of their regime. They also develop asymptotically valid inference for a parameter similar to improvement with business-as-usual as the random (see our Section 3.3), an idea we extend. Within the literature, their work is the closest conceptually to ours. Gunter et al. (2011b) develop a stepwise approach to variable selection and in Gunter et al. (2011a) compare it to stepwise regression. Rather than using a traditional sum-of-squares metric, the authors’ method compares the estimated mean response, or “value,” of the optimal policy for the models considered, a concept we make use of in Section 3. Imai and Ratkovic (2013) use a modified Support Vector Machine with LASSO constraints to select the variables useful in an optimal regime when the response is binary. van der Laan and Luedtke (2015) uses a loss-based super-learning approach with cross-validation.

Also important within the area of treatment regime estimation, but not explored in this paper, is the estimation of dynamic treatment regimes (DTRs). DTRs constitute a set of decision rules, estimated from many experimental and longitudinal intervals. Each regime is intended to produce the highest mean response over that time interval. Naturally, the focus is on optimal DTRs—the decision rules which provide the highest mean response. Murphy (2003) and Robins (2004) develop two influential approaches based on regret functions and nested mean models respectively. Moodie et al. (2007) discuss the relationship between the two while Moodie and Richardson (2009) and Chakraborty et al. (2010) present approaches for mitigating biases (the latter also fixes biases in model parameter estimation stemming from their non-regularity in SMART trials). Robins et al. (2008) focus on using observational data and optimizing the time for administering the stages—the “when to start”—within the DTR. Orellana et al. (2010) develop a different approach for estimating optimal DTRs based on marginal structural mean models. Henderson et al. (2010) develop optimal DTR estimation using regret functions and also focus on diagnostics and model checking. Barrett et al. (2014) develop a doubly robust extension of this approach for use in observational data. Laber et al. (2014) demonstrate the application of set-valued DTRs that allow balancing of multiple possible outcomes, such as relieving symptoms or minimizing patient side effects. Their approach produces a subset of recommended treatments rather than a single treatment. Also, McKeague and Qian (2014) estimate treatment regimes from functional predictors in RCTs to incorporate biosignatures such as brain scans or mass spectrometry.

Many of the procedures developed for estimating DTRs have roots in reinforcement learning. Two widely used methods are Q-learning Murphy (2005a) and A-learning (see Schulte et al., 2014 for an overview of these concepts). One well-noted difficulty with Q-learning and A-learning are their susceptibility to model misspecification. Consequently, researchers have begun to focus on “robust” methods for DTR estimation. Zhang et al. (2013) extend the doubly robust augmented inverse probability weighted method (Zhang et al., 2012a) by considering multiple binary treatment stages.

Many of the methods mentioned above can be extended to censored survival data. Zhao et al. (2015) describe a computationally efficient method for estimating a treatment regimes that maximizes mean survival time by extending the weighted learning inverse probability method. This method is doubly robust; it is protected from model misspecification if either the censoring model or the survival model is correct. Additionally, methods for DTR estimation can be extended. Goldberg and Kosorok (2012) extend Q-learning with inverse-probability-of-censoring weighting to find the optimal treatment plan for individual patients, and the method allows for flexibility in the number of treatment stages.

It has been tempting, when creating these treatment regime models, to directly employ them to predict the differential response of individuals among different treatments. This is called in the literature “heterogeneous treatment effects models” or “individualized treatment rules” and there is quite a lot of interest in it.

Surprisingly, methods designed for accurate estimation of an overall conditional mean of the response may not perform well when the goal is to estimate these individualized treatment rules. Qian and Murphy (2011) propose a two-step approach to estimating “individualized treatment rules” based on single-stage randomized trials using $\ell_{1}$-penalized regression while Lu et al. (2013) use quadratic loss which facilitates variable selection. Rolling and Yang (2014) develop a new form of cross-validation which chooses between different heterogeneous treatment models.

One current area of research in heterogeneous effect estimation is the development of algorithms that can be used to create finer and more accurate partitions. Kallus (2017) presents three methods for the case of observational data: greedily partitioning data to find optimal trees, bootstrap aggregating to create a “personalization forest” a la Random Forests, and using the tree method coupled with mixed integer programming to find the optimal tree. Lamont et al. (2018) build on the prior methods of parametric multiple imputation and recursive partitioning to estimate heterogeneous treatment effects and compare the performance of both methods. This estimation can be extended to censored data. Henderson et al. (2020) discuss the implementation of Bayesian Additive Regression Trees for estimating heterogeneous effects which can be used for continuous, binary and censored data. Ma et al. (2019) proposes a Bayesian predictive method that integrates multiple sources of biomarkers.

One major drawback of many of the approaches in the literature reviewed is their significant difficulty evaluating estimator performance. Put another way, given the complexity of the estimation procedures, statistical inference is very challenging. Many of the approaches require that the proposed model be correct. There are numerous applications in the biomedical sciences for which this assumption is neither credible nor testable in practice. For example, Evans and Relling (2004) consider pharmacogenomics, and argue that as our understanding of the genetic influences on individual variation in drug response and side-effects improves, there will be increased opportunity to incorporate genetic moderators to enhance personalized treatment. But we will ever truly understand the endpoint model well enough to properly specify it? Further, other biomarkers (e.g. neuroimaging) of treatment response have begun to emerge, and the integration of these diverse moderators will require flexible approaches that are robust to model misspecification (McGrath et al., 2013). How will the models of today incorporate important relationships that can be anticipated but have yet to be identified? Further, many proposed methods employ non-parametric models that use the data to decide which internal parameters to fit and then in turn estimates these internal parameters. Thus a form of model selection that introduces difficult inferential complications (see Berk et al., 2013b).

At the very least, therefore, there should be an alternative inferential framework for evaluating treatment regimes that do not require correct model specification (and thereby obviating the need for model checking and diagnostics) nor knowledge of unmeasured characteristics (see discussion in Henderson et al., 2010) accompanied by easy-to-use software. This is the modest goal herein.

## 3 Methods

This work seeks to be didactic and thus carefully explains the extant methodology and framework (Section 3.1), data inputs (Section 3.2), estimation (Section 3.4) and a procedure for inference (Section 3.5). For those familiar with this literature, these sections can be skipped. Our methodological contributions then are to 1) employ out-of-sample validation to (Section 3.4) specifically to 2) *improvement*, the metric defined as the difference in how patients allocated via personalized model fare in their outcome and a patients allocated via business-as-usual model fare in their outcome (Section 3.3) and 3) to extend this validation methodology to binary and survival endpoints (Sections 3.4.2 and 3.4.3). Table 1 serves as a guide to the main notation used in this work, organized by section.

**TABLE 1:** A compendium of the main notation in our methodology by section.

Notation	Description
Framework (Section 3.1)	
*Y*	The random variable (r.v.) for the outcomes for the subjects
X, X	The r. v. for the observed measurements for the subjects, its support
*A*	The r. v. for the treatment
$T_{1},T_{2}$	The two treatments, shorthand for their codes, zero and one
*d* or d[f]	The decision function; this function maps observed measurements to treatment
*V* or V[d]	The value of the decision function, the average outcome over all patients if this decision is used to allocate treatment
$d^{\text{*}}$	The unknown optimal decision function i.e. the one with highest *V*
$d_{0}$	A naive, baseline, business-as-usual or null decision function
$\mu_{I_{0}}$	The unknown improvement of *d* over $d_{0}$; it is the difference of values
The RCT data (Section 3.2.1)	
*n*	The number of subjects in the randomized comparative trial (RCT)
*p*	The number of measurements assessed on each subject
$x_{i}$	The vector of *p* measurements for the ith subject
$x_{i,j}$	The jth measurement for the ith subject
X	The n×p matrix of all measurements for all subjects
A	The vector of treatments for all the *n* subjects
y	The vector of outcomes for all the *n* subjects
The response model (Section 3.2.2)	
*f*	The function that relates the *p* measurements and *A* to the response
$U_{i}$	The r. v. for the unknown covariates for subject *i*
$\xi \left(x_{i},A_{i},U_{i}\right)$	The function that computes misspecification in the response
${ℰ}_{i}$	The r. v. for irreducible noise for the ith subject
$\beta_{j}$	The linear coefficient for the *j*th measurement when A=0
$\gamma_{j}$	The additional linear coefficient for the *j*th measurement when A=1
Out of sample estimation and validation (Section 3.4)	
$X_{\text{train}},y_{\text{train}}$	The subset of the data used to create the fit of *f*
$X_{\text{test}},y_{\text{test}}$	The subset of the data used to validate the fit of *f*
$\hat{f}$, $\hat{d}$, $\hat{V}$, ${\hat{I}}_{0}$	The finite-sample estimates of *f*, *d*, *V*, $\mu_{I_{0}}$
${\bar{y}}_{set}$	The arithmetic average of $\left\{y_{i}:i\in \mathrm{set}\right\}$
${\hat{\beta }}_{j}$, ${\hat{\gamma }}_{j}$	The finite-sample estimates of $\beta_{j}$, $\gamma_{j}$
Inference (Section 3.5)	
*B*	The number of bootstrap samples
$\tilde{X},\tilde{y}$	A sample of the rows of X,y with replacement
${\tilde{I}}_{0,b}$	The *b*th estimate of $\mu_{I_{0}}$ in the bootstrap
*α*	The size of the hypothesis test
Personalization of future subjects’ treatments (Section 3.6)	
$x_{*}$	A future subject (not part of the RCT)

### 3.1 Conceptual Framework

We imagine a set of random variables having a joint probability distribution that can be also be viewed as a population from which data could be randomly and independently realized. The population can also be imagined as all potential observations that could be realized from the joint probability distribution. Either conception is consistent with our setup.

A researcher chooses one of the random variables to be the response *Y* which could be continuous, binary or survival (with a corresponding variable that records its censoring, explained later). We assume without loss of generality that a higher-valued outcome is better for all individuals. Then, one or more of the other random variables are covariates X∈X. At the moment, we do not distinguish between observed and unobserved covariates but we will later. There is then a conditional distribution P(Y|X) whose conditional expectation function E[Y|X] constitutes the population response surface. No functional forms are imposed on the conditional expectation function and thus it is allowed to be nonlinear with interactions among the covariates which is the case in artificial intelligence procedures (machine learning).

All potential observations are hypothetical study subjects. Each can be exposed to a random treatment denoted A ∈{0,1} where zero codes for the first experimental condition also equivalently referred to as $T_{1}$ (which may be considered the “control” or “comparison” condition) and one codes for another experimental condition equivalently referred to as $T_{2}$. We make the standard assumption of no interference between study subjects, which means that the outcome for any given subject is unaffected by the interventions to which other subjects are randomly assigned (Cox, 1958) and outcomes under either condition can vary over subjects (Rosenbaum, 2002, Section 2.5.1). In short, we employ the conventional Neyman-Rubin approach (Rubin, 1974) but treat all the data as randomly realized (Berk et al., 2013a).

A standard estimation target in RCTs is the population average treatment effect (PATE), defined here as E[Y|A=1]−E[Y|A=0], the difference between the population expectations. That is, the PATE is defined as the difference in mean outcome were all subjects exposed to $T_{2}$ or alternatively were all exposed to $T_{1}$. In a randomized controlled trial, the PATE is synonymous with the overall efficacy of the treatment of interest and it is almost invariably the goal of the trial (Zhao and Zeng, 2013).

For personalization, we want to make use of any association between *Y* and X. For the hypothetical study subjects, there is a conditional population response surface E[Y|X,A=1] and another conditional population response surface E[Y|X,A=0], a key objective being to exploit the difference in these response surfaces for better treatment allocation. The typical approach is to create a deterministic *individualized treatment decision rule d* that takes an individual’s covariates and maps them to a treatment. We seek d:X→{0,1} based on knowledge of the differing conditional population response surfaces. The rule is sometimes called an *allocation procedure* because it determines which treatment to allocate based on measurements made on the individual. To compare different allocation procedures, our metric is the expectation of the outcome *Y* using the allocation procedure *d* averaged over all subjects X. Following the notation of Qian and Murphy (2011), we denote this expectation as the value of the decision rule

$$V\left[d\right]:=E_{X,A}^{d}\left[Y\right]≜\int_{X}\left(\sum_{a\in \left\{0,1\right\}}\left(\int_{\mathbb{R}}yf_{Y|X,A}\left(y,x,a\right)dy\right)1_{a=d\left(x\right)}\right)f_{X}\left(x\right)\text{d}x.$$

Although the integral expression appears complicated, when unpacked it is merely an expectation of the response averaged over X, the space of all patients characteristics. When averaging over X, different treatments will be recommended based on the rule, i.e. a=d(x), and that in turn will modify the density of the response, $f_{\mathrm{Y|X}}$. Put another way, V[d] is the mean patient outcome when personalizing each patient’s treatment.

We have considered all covariates to be random variables because we envision *future* patients for whom an appropriate treatment is required. Ideally, their covariate values are realized from the same joint distribution as the covariate values for the study subjects, an assumption that is debated and discussed in the concluding section.

In addition, we do not intend to rely on estimates of the two population response surfaces. As a practical matter, we will make do with a population response surface approximation for each. No assumptions are made about the nature of these approximations and in particular, how well or poorly either population approximation corresponds to the true conditional response surfaces.

Recall that much of the recent literature has been focused on finding the optimal rule, $d^{*}≜\arg\max_{d}\left\{V\left[d\right]\right\}$. Although this is an admirable ideal (as in Qian and Murphy 2011), our goals here are more modest. We envision an imperfect rule *d* far from $d^{*}$, and we wish to gauge its performance relative to the performance of another rule $d_{0}$, where the “naught” denotes a business-as-usual allocation procedure, sometimes called “standard of care”. Thus, we define the population value improvement $\mu_{I_{0}}$ as the value of *d* minus the value of $d_{0}$,

$$\mu_{I_{0}}≜V\left[d\right]-V\left[d_{0}\right]=E_{X,A}^{d}\left[Y\right]-E_{X,A}^{d_{0}}\left[Y\right],$$ (1)

which is sometimes called “benefit” in the literature. Since our convention is that higher response values are better, we seek large, positive improvements that translate to better average performance (as measured by the response). Note that this is a natural measure when *Y* is continuous. When *Y* is incidence or survival, we redefine $\mu_{I_{0}}$ (see Sections 3.4.2 and 3.4.3).

The metric $\mu_{I_{0}}$ is not standard in the literature but we strongly believe it to be the natural metric for personalization following Kallus (2017). There are many other such metrics beyond value and improvement. For example, Ma et al. (2019) uses three 1) the expected number of subjects misassigned to their optimal treatment, 2) exepected gain or loss in treatment utility and 3) the expected proportion where the model correctly predicted the response which is useful only in the binary response case which we address later.

### 3.2 Our Framework’s Required Inputs

Our method depends on two inputs 1) access to RCT data and 2) either a prespecified parametric model f(x,A;θ) for the population approximation of the true response surfaces or an explicit *d* function. If we prespecified *f*, we then use the RCT data to estimate parameters of the model $\hat{\theta }$, and the estimates are embedded in the estimated model, $\hat{f}$. This model estimate permits us, in turn, to construct an estimated decision rule $\hat{d}$ and an estimate of the improved outcomes future subjects will experience (explained later in Section 3.4). We assume that the model *f* is specified before looking at the data. “Data snooping” (running our method, checking the *p*-value, changing the model *f* and running again) fosters overfitting and can introduce serious estimation bias, invalidating our confidence intervals and statistical tests (Berk et al., 2013b).

#### 3.2.1 The RCT Data

Our procedure strictly requires RCT data to ensure there is a causal effect of the heterogeneous parameters. Much of the research discussed in the background (Section 2) applies in the case of observational data. We realize this limits the scope of our proposal. The RCT data must come from an experiment undertaken to estimate the PATE for treatments $T_{1}$ and $T_{2}$ for a diagnosis of a disease of interest. $T_{1}$ and $T_{2}$ are the same treatments one would offer to future subjects with the same diagnosis.

There are *n* subjects each with *p* covariates which are denoted for the *i*th subject as $x_{i}≜\left[x_{i1},x_{i2},\ldots ,x_{ip}\right]$. Because these covariates will be used to construct a decision rule applied with future patients in clinical settings, the $x_{i}$’s in the RCT data must be the same covariates measured for new subjects. Thus, such characteristics such as the site of treatment (in a multi-center trial) or the identification of the medical practitioner who treated each subject or hindsight-only variables are not included.

We assume the outcome measure of interest $y_{i}$ is assessed once per subject. Aggregating all covariate vectors, binary allocations and responses rowwise, we denote the full RCT data as the column-bound matrix [X,A,y]. In practice, missing data can be imputed (in both the RCT data and the future data), but herein we assume complete data.

We will be drawing inference to a patient population beyond those who participated in the experiment. Formally, new subjects must be sampled from that same population as were the subjects in the RCT. In the absence of explicit probability sampling, the case would need to be made that the model can generalize. This requires subject-matter expertise and knowledge of how the study subjects were recruited.

#### 3.2.2 The Model for the Response Based on Observed Measurements

The decision rule *d* is a function of x through *f* and is defined as

$$d\left[f\left(x\right)\right]≜\underset{A\in \left\{0,1\right\}}{\arg\max}f\left(x,A\right)=1_{f\left(x,1\right)-f\left(x,0\right)}.$$ (2)

As in Berk et al. (2014), we assume the model *f* provided by the practitioner to be an approximation using the available information,

$$Y_{i}=\underset{E\left[Y_{i}|X_{i},U_{i},A_{i}\right]}{\underset{︸}{f\left(X_{i},A_{i}\right)+\xi \left(X_{i},U_{i},A_{i}\right)}}+{ɛ}_{i},$$ (3)

where *f* differs from the true response expectation by a term dependent on U, the unobserved information. The last term ${ɛ}_{i}$ is the irreducible noise around the true conditional expectations and is taken to be independent and identically distributed, mean-centered and uncorrelated with the covariates. Even in the absence of ${ɛ}_{i}$, *f* will always differ from the true conditional expectation function by $\xi_{i}\left(X_{i},U_{i},A_{i}\right)$, which represents model misspecification (Box and Draper, 1987, Chapter 13).

We wish only to determine whether an estimate of *f* is *useful* for improving treatment allocation for future patients (that are similar to the patients in the RCT) and do not expect to recover the optimal allocation rule $d^{*}$ which requires the unseen U. Further, we do not concern ourselves with substantive interpretations associated with any of the *p* covariates, a goal of future research. Thus, our method is robust to model misspecification by construction.

What could *f* look like in practice? Assume a continuous response (binary and survival are discussed later) and consider the conventional linear regression model with first order interactions. Much of the literature we reviewed in Section 2 favored this class of models. We specify a linear model containing a subset of the covariates used as main effects and a possibly differing subset of the covariates to be employed as first order interactions with the treatment indicator, $\left\{x_{1^{\prime }},\ldots ,x_{p^{\prime }}\right\}\subset \left\{x_{1},\ldots ,x_{p}\right\}$, selected using domain knowledge:

$$f\left(x_{i1},A_{i}\right)=\beta_{0}+\beta_{1}x_{1}+\cdots +\beta_{p}x_{p}+A_{i}\left(\gamma_{0}+\gamma_{1^{\prime }}x_{1^{\prime }}+\cdots +\gamma_{p^{\prime }}x_{p^{\prime }}\right).$$ (4)

These interactions induce heterogeneous effects between $T_{1}$ and $T_{2}$ for a subject x in a very interpretable way: d[f(x)]=1 when $\gamma_{0}+\gamma_{1^{\prime }}x_{1^{\prime }}+\cdots +\gamma_{p^{\prime }}x_{p^{\prime }}>0$ and 0 otherwise. The γ’s are the critical component of the model if there are systematic patient-specific differences between the interventions. Thereby, *d* varies over different points in X space. Note that rules derived from this type of conventional model also have the added bonus as being interpretable as a best linear approximation of the true relationship.

We stress that our models are *not* required to be of this form, but we introduce them here mostly for familiarity and pedagogical simplicity. There are times when this linear model will perform terribly even if $\left\{x_{1^{\prime }},\ldots ,x_{p^{\prime }}\right\}$ are the correct moderating variables. For a non-linear example, see Zhao and Zeng (2013), Figure 1, right. Although this model is the default implementation, the user can specify any model desired in the software. This will be discussed in Section 5.

**FIGURE 1:**
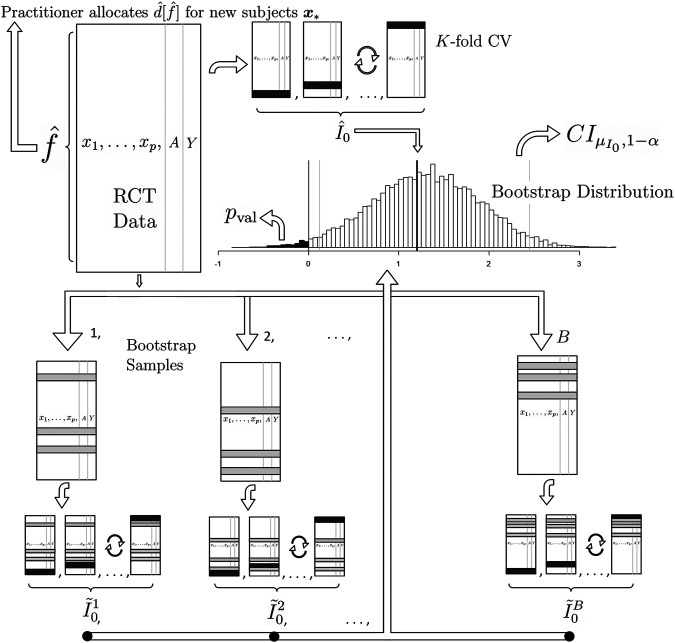
A graphical illustration of (1) our proposed method for estimation and (2) our proposed method for inference on the population mean improvement of an allocation procedure and (3) our proposed future allocation procedure (top left of the illustration). To compute the best estimate of the improvement ${\hat{I}}_{0}$, the RCT data goes through the *K*-fold cross validation procedure of Section 3.4 (depicted in the top center). The black slices of the data frame represent the test data. To draw inference, we employ the non-parametric bootstrap procedure of Section 3.5 by sampling the RCT data with replacement and repeating the *K*-fold CV to produce ${\hat{I}}_{0}^{1},{\hat{I}}_{0}^{2},\ldots ,{\hat{I}}_{0}^{B}$ (bottom). The gray slices of the data frame represent the duplicate rows in the original data due to sampling with replacement. The confidence interval and significance of $H_{0}:\mu_{I_{0}}\leq 0$ is computed from the bootstrap distribution (middle center). Finally, the practitioner receives $\hat{f}$ which is built with the complete RCT data (top left).

We also stress that although the theory for estimating linear models’ coefficients (as well as those for logistic regression and Weibull regression) is well-developed, we are not interested in inference for these coefficients in this work as our goal is only estimation and inference for overall usefulness of the personalization scheme, i.e. the unknown parameter $\mu_{I_{0}}$. This will become clear in the next few sections.

### 3.3 Other Allocation Procedures

Although $d_{0}$ can be any allocation rule, for the purposes of the paper, we examine only two “business-as-usual” allocation procedures (others are discussed as extensions in Section 6). The first we call **random** denoting the allocation where the patient receives $T_{1}$ or $T_{2}$ with a fair coin flip, probability 50%. This serves as a baseline or “straw man” but nevertheless an important standard—the personalization model should be able to provide better patient outcomes than a completely random allocation.

The second business-as-usual procedure we call **best**. This procedure gives all patients the better of the two treatments as determined by the comparison of the sample average for all subjects who received $T_{1}$ denoted ${\bar{y}}_{T_{1}}$ and the sample average of all subjects who received $T_{2}$ denoted ${\bar{y}}_{T_{2}}$. This is used as the default in many frameworks for example Kang et al. (2014). We consider beating this procedure the gold standard in proof that the personalization truly works as practitioners most often employ the current best known treatment. However, some consider this comparison conservative (Brinkley et al., 2010, Section 7) and the next section will describe why it is statistically conservative as we lose sample size when demanding this comparison. Due to this conservativeness, barring conclusive evidence that either $T_{1}$ or $T_{2}$ is superior, the random procedure should be the standard of comparison. This case is not infrequent in RCTs which feature negative comparison results, the case in our clinical trial example of Section 4.3.

### 3.4 Estimating the Improvement Scores

#### 3.4.1 For a Continuous Response

How well do unseen subjects with treatments allocated by *d* do on average compared to the same unseen subjects with treatments allocated by $d_{0}$? We start by computing the estimated improvement score, a sample statistic given by

$${\hat{I}}_{0}≜\hat{V}\left[\hat{d}\right]-\hat{V}\left[\hat{d_{0}}\right],$$ (5)

where $\hat{d}$ is an estimate of the rule *d* derived from the population response surface approximation, $\hat{V}$ is an estimate of its corresponding value *V* and ${\hat{I}}_{0}$ is an estimate of the resulting population improvement $\mu_{I_{\text{0}}}$ (Eq. 1). The $\hat{d_{\text{0}}}$ notation indicates that sometimes the competitor $d_{\text{0}}$ may have to be estimated from the data as well. For example, the allocation procedure **best** must be calculated by using the sample average of the responses for both $T_{1}$ and $T_{2}$ in the data.

In order to properly estimate $\mu_{I_{\text{0}}}$, we use cross-validation (Hastie et al., 2013, Chapter 7.10). We split the RCT data into two disjoint subsets: training data with $n_{\text{train}}$ of the original *n* observations $\left[X_{\text{train}},y_{\text{train}}\right]$ and testing data with the remaining $n_{\text{test}}=n-n_{\text{train}}$ observations $\left[X_{\text{test}},y_{\text{test}}\right]$. Then ${\hat{f}}_{\text{train}}$ can be fit using the training data to construct $\hat{d}$ via Eq. 2. Performance of $\hat{d}$ as calculated by Equation 5, is then evaluated on the test data. Hastie et al. (2013) explain that a single train-test split yields an estimate of the “performance” of the procedure on future individuals conditional on $\left[X_{\text{train}},y_{\text{train}}\right]$, the “past”. Thus, the ${\hat{I}}_{0}$ statistic defined in Eq. 5 computed using $\left[X_{\text{test}},y_{\text{test}}\right]$ can provide an honest assessment of improvement (i.e. immune to overfitting in $\hat{f}$) who are allocated using our proposed methodology compared to a baseline business-as-usual allocation strategy (Faraway, 2016). This can be thought of as employing a replicated trial, often required in drug development programs, which separates rule construction (in-sample) from rule validation (out-of-sample) as recommended by Rubin and van der Laan (2012). Note that this comes at a cost of more sample variability (as now our estimate will be based on the test subset with a sample size much smaller than *n*). Our framework and software is the first to provide user-friendly out-of-sample validation for the overall utility of personalized medicine models as a native feature.

Given the estimates $\hat{d}$ and $\hat{d_{0}}$, the question remains of how to explicitly compute $\hat{V}$ for subjects we have not yet seen in order to estimate ${\hat{I}}_{0}$. That is, we are trying to estimate the expectation of an allocation procedure over covariate space X.

Recall that in the test data, our allocation prediction $\hat{d}$(**x**_*i*_) is the binary recommendation of $T_{1}$ or $T_{2}$ for each $x_{\text{test},i}$. If we recommended the treatment that the subject actually was allocated in the RCT, i.e. $\hat{d}$(***x***+_*i*_) = *A*_*i*_, we consider that subject to be “lucky”. We define lucky in the sense that by the flip of the coin, the subject was randomly allocated to the treatment that our model-based allocation procedure estimates to be the better of the two treatments.

The average of the lucky subjects’ responses should estimate the average of the response of new subjects who are allocated to their treatments based on our procedure *d* and this is the estimate of $\hat{V}\left[\hat{d}\right]$ we are seeking. Because the x’s in the test data are assumed to be sampled randomly from population covariates, this sample average estimates the expectation over X, i.e. $E_{X,A}^{d}\left[Y\right]$ conditional on the training set. In order to make this concept more clear, it is convenient to consider Table 2, a 2×2 matrix which houses the sorted entries of the out-of-sample $y_{\text{test}}$ based on the predictions, the $\hat{d}$(***x***_*i*_)’s.

**TABLE 2:** The elements of $y_{\text{test}}$ cross-tabulated by their administered treatment $A_{i}$ and our model’s estimate of the better treatment $\hat{d}$(**x**_*i*_).

	$\hat{d}$(**x**_*i*_) = 0	$\hat{d}$(**x**_*i*_) = 1
*A*_*i*_ = 0	P	Q
*A*_*i*_ = 1	R	S

The diagonal entries of sets *P* and *S* contain the “lucky” subjects. The notation ${\bar{y}}_{\cdot }$ indicates the sample average among the elements of $y_{\text{test}}$ specified in the subscript located in the cells of the table.

How do we compute $\hat{V}\left[{\hat{d}}_{0}\right]$, the business-as-usual procedure? For **random**, we simply average all of the $y_{\text{test}}$ responses; for **best**, we average the $y_{\text{test}}$ responses for the treatment group that has a larger sample average. Thus, the sample statistics of Eq. 5 can be written as

$${\hat{I}}_{\text{random}}≜{\bar{y}}_{P\cup S}-{\bar{y}}_{\text{test}},$$ (6)

$${\hat{I}}_{\text{best}}≜{\bar{y}}_{P\cup S}-\{\begin{array}{cc} {\bar{y}}_{P\cup Q}, & \text{when}\;{\bar{y}}_{P\cup Q}\geq {\bar{y}}_{R\cup S},\\ {\bar{y}}_{R\cup S}, & \text{when}\;{\bar{y}}_{P\cup Q}<{\bar{y}}_{R\cup S}. \end{array}$$ (7)

Note that the plug-in estimate of value $\hat{V}\left[\hat{d}\right]={\bar{y}}_{P\cup S}$ is traditional in the personalized medicine literature. For example, in Kallus (2017), Corollary 3 it is written as

$$\hat{V}\left[\hat{d}\right]:=\frac{\sum_{i=1}^{n}Y_{i}1_{\hat{d}\left(x_{i}\right)=A_{i}}}{\sum_{i=1}^{n}1_{\hat{d}\left(x_{i}\right)=A_{i}}}.$$ (8)

There is one more conceptual point. Recall that the value estimates $\hat{V}\left[\cdot \right]$ are conditional on the training set. This means they do not estimate the unconditional $E_{X,A}^{d}\left[Y\right]$. To address this, Hastie et al. (2013), Chapter 7 recommend that the same procedure be performed across many different mutually exclusive and collectively exhaustive splits of the full data. This procedure of building many models is called “*K*-fold cross-validation” (CV) and its purpose is to integrate out the effect of a single training set to result in the unconditional estimate of generalization. This is “an alternative approach … [that] for simplicity … [was not] consider [ed] … further” in the previous investigation of Chakraborty et al. (2014), page 5.

In practice, how large should the training and test splits be? Depending on the size of the test set relative to the training set, CV can trade bias for variance when estimating an out-of-sample metric. Small training sets and large test sets give more biased estimates since the training set is built with less data than the *n* observations given. However, large test sets have lower variance estimates since they are composed of many examples. There is no consensus in the literature about the optimal training-test split size (Hastie et al., 2013, page 242) but 10-fold CV is a common choice employed in many statistical applications and provides for a relatively fast algorithm. In the limit, *n* models can be created by leaving each observation out, as done in DeRubeis et al. (2014). In our software, we default to 10-fold cross validation but allow for user customization.

This estimation procedure outlined above is graphically illustrated in the top of Figure 1. We now extend this methodology to binary and survival endpoints in the next two sections.

#### 3.4.2 For a Binary Response

In the binary case, we let *V* be the expected probability of the positive outcome under *d* just like in Kang et al. (2014). We consider three improvement metrics 1) the probability difference, 2) the risk ratio and 3) the odds ratio:

$$\begin{array}{l} \left(\text{a}\right)\mu_{I_{0}}≜V\left[d\right]-V\left[d_{0}\right],\\ \left(\text{b}\right)\mu_{I_{0}}≜\frac{V\left[d\right]}{V\left[d_{0}\right]},\\ \left(\text{c}\right)\mu_{I_{0}}≜\frac{V\left[d\right]/1-V\left[d\right]}{V\left[d_{0}\right]/1-V\left[d_{0}\right]}. \end{array}$$

and the estimate of all three (the probability difference, the risk ratio and the odds ratio) is found by placing hats on each term in the definitions above (all *V*’s, *d*’s and $d_{0}$’s).

Following the example in the previous section we employ the analogous model, a logistic linear model with first order treatment interactions where the model *f* now denotes the probability of the positive outcome y=1,

$$f\left(x_{i1},A_{i}\right)=\log\text{it}\left(\beta_{0}+\beta_{1}x_{1}+\cdots +\beta_{p}x_{p}+A_{i}\left(\gamma_{0}+\gamma_{1^{\prime }}x_{1^{\prime }}+\ldots +\gamma_{p^{\prime }}x_{p^{\prime }}\right)\right).$$ (9)

This model, fit via maximum likelihood numerically (Agresti, 2018), is the default in our software implementation. Here, higher probabilities of success imply higher logit values so that algebraically we have the same form of the decision rule estimate, $\hat{d}\left[\hat{f}\left(x\right)\right]=1$ when ${\hat{\gamma }}_{0}+{\hat{\gamma }}_{1^{\prime }}x_{1^{\prime }}+\cdots$ $+{\hat{\gamma }}_{p^{\prime }}x_{p^{\prime }}>0$.

If the risk ratio or odds ratio improvement metrics are desired, Eqs. 6, 7 are modified accordingly but otherwise estimation is then carried out the same as in the previous section.

#### 3.4.3 For a Survival Response

Survival responses differ in two substantive ways from continuous responses: 1) they are always non-negative and 2) some values are “censored” which means it appropriates the value of the last known measurement but it is certain that the true value is greater. The responses y are coupled with this censoring information c, a binary vector of length *n* where the convention is to let $c_{i}=0$ to indicate that $y_{i}$ is censored and thus set equal to its last known value.

To obtain $\hat{d}$, we require a survival model. For example purposes here we will assume the exponential regression model (the exponentiation enforces the positivity of the response values) with the usual first order treatment interactions,

$$f\left(x_{i1},A_{i}\right)=\exp\left(\beta_{0}+\beta_{1}x_{1}+\cdots +\beta_{p}x_{p}+A_{i}\left(\gamma_{0}+\gamma_{1^{\prime }}x_{1^{\prime }}+\cdots +\gamma_{p^{\prime }}x_{p^{\prime }}\right)\right).$$ (10)

Under the exponential model, the convention is that the noise term ɛ is multiplicative instead of additive, $Y_{i}=f\left(x_{i1},A_{i}\right){ɛ}_{i}$. Note that at this step, a fully parametric model is needed; the non-parametric Kaplan-Meier or the semi-parametric Cox proportion hazard model are insufficient as we need a means of explicitly estimating E[Y | X,A] for all values of *X* and both values of *A*.

Moreso than for continuous and incidence endpoints, parameter estimation is dependent on the choice of error distribution. Following Hosmer and Lemeshow (1999), a flexible model is to let $\ln\left({ɛ}_{1}\right),\ldots ,\ln\left({ɛ}_{n}\right){∼}^{\text{iid}}$ $\text{Gumbel}\left(0,\sigma^{2}\right)$, implying the popular Weibull model for survival (and the default in our software). As was the case previously, the user is free to choose whatever model they wish. The $\beta_{j}$’s, $\gamma_{j}$’s and the nuisance scale parameter $\sigma^{2}$ are fit using maximum likelihood taking care to ensure the correct contributions of censored and uncensored values. Similar to the case of logistic regression, the likelihood function does not have a closed form solution and must be approximated numerically.

Some algebra demonstrates that the estimated decision rule is the same as those above, i.e. $\hat{d}\left[\hat{f}\left(x\right)\right]=1$ when ${\hat{\gamma }}_{0}+{\hat{\gamma }}_{1^{\prime }}x_{1^{\prime }}+\cdots +{\hat{\gamma }}_{p^{\prime }}x_{p^{\prime }}>0$. In other words, the subject is given the treatment that yields the longest expected survival.

Subjects are then sorted in cells like Table 2 but care is taken to keep the corresponding $c_{i}$ values together with their paired $y_{i}$ values following Yakovlev et al. (1994). At this point, we need to specify analogous computations to Eqs. 6, 7 that are sensitive to the fact that many $y_{i}$ values are censored (The sample averages $\bar{y}$ obviously cannot be employed here because it ignores this censoring).

Of course we can reemploy a new Weibull model and define improvement as we did earlier as the difference in expectations (Eq. 1). However, there are no more covariates needed at this step as all subjects have been sorted based on $\hat{d}\left(x\right)$. Thus, there is no reason to require a parametric model that may be arbitrarily wrong.

For our default implementation, we have chosen to employ the difference of the Kaplan-Meier median survival statistics here because we intuitively feel that a non-parametric estimate makes the most sense. Once again, the user is free to employ whatever they feel is most appropriate in their context. Given this default, please note that the improvement measure of Eq. 1 is no longer defined as the difference in survival expectations, but now the difference in survival medians. This makes our framework slightly different in the case of survival endpoints.

### 3.5 Inference for the Population Improvement Parameter

Regardless of the type of endpoint, the ${\hat{I}}_{0}$ estimates are drawn from an elaborate estimator whose sampling distribution is not available in closed form. We can employ the nonparametric bootstrap to obtain an asymptotic estimate of its sampling variability, which can be used to construct confidence intervals and testing procedures (Efron and Tibshirani, 1994).

In the context of our proposed methodology, the bootstrap procedure works as follows for the target of inference $\mu_{I_{0}}$. We take a sample with replacement from the RCT data of size *n* denoted with tildes: $\left[\tilde{X},\tilde{y}\right]$. Using the 10-fold CV procedure described at the end of Section 3.4, we create an estimate ${\tilde{I}}_{0}$. We repeat the resampling of the RCT data and the recomputation of ${\tilde{I}}_{0}$ *B* times where *B* is selected for resolution of the confidence interval and significance level of the test. In practice we found B=3000 to be sufficient, so we leave this as the default in our software implementation. Because the *n*’s of usual RCTs are small, and the bootstrap is embarrassingly parallelizable, this is not an undue computational burden.

In this application, the bootstrap approximates the sampling of many RCT datasets. Each $\tilde{I}$ that is computed corresponds to one out-of-sample improvement estimate for a particular RCT dataset drawn from the population of RCT datasets. We stress again that the frequentist confidence intervals and tests that we develop for the improvement measure do *not* constitute inference for a new individual’s improvement, it is inference for the average improvement for future subjects vs. **random** allocation, $\mu_{I_{0}}$.

To create a 1−α level confidence interval, first sort the $\left\{{\tilde{I}}_{0,1},\ldots ,{\tilde{I}}_{0,B}\right\}$ by value, and then report the values corresponding to the empirical α/2 and 1−α/2 percentiles. This is called the “percentile method.” “Although this direct equation of quantiles of the bootstrap sampling distribution with confidence limits may seem initially appealing, its “rationale is somewhat obscure” (Rice, 1994, page 272). There are other ways to generate asymptotically valid confidence intervals using bootstrap samples but there is debate about which has the best finite sample properties. We have also implemented the “basic method” (Davison and Hinkley, 1997, page 194) and the bias-corrected “$BC_{a}$ method” of Efron (1987) that DiCiccio and Efron (1996) claim performs an order of magnitude better in accuracy than the percentile method. Implementing other confidence interval methods for the bootstrap may be useful future work.

If a higher response is better for the subject, we set $H_{0}:\mu_{I_{0}}\leq 0$ and $H_{a}:\mu_{I_{0}}>0$. Thus, we wish to reject the null hypothesis that our allocation procedure is at most as useful as a naive business-as-usual procedure. To obtain an asymptotic *p* value based on the percentile method, we tally the number of bootstrap sample $\tilde{I}$ estimates below 0 and divide by *B*. This bootstrap procedure is graphically illustrated in the bottom half of Figure 1 and the bootstrap confidence interval and *p* value computation is illustrated in the center. Note that for incidence outcomes where the improvement is defined as the risk ratio or odds ratio, we use $H_{0}:\mu_{I_{0}}\leq 1$ and $H_{a}:\mu_{I_{0}}>1$ and tally the number of $\tilde{I}$ estimates below 1.

We would like to stress once again that we are not testing for qualitative interactions—the ability of a covariate to “flip” the optimal treatment for subjects. Tests for such interactions would be hypothesis tests on the *γ* parameters of Eqs. 4, 9, 10, which assume model structures that are not even required for our procedure. Qualitative interactions are controversial due to model dependence and entire tests have been developed to investigate their significance. In the beginning of Section 2 we commented that most RCTs are not even powered to investigate these interactions. “Even if an optimal personalized medicine rule [based on such interactions] can provide substantial gains it may be difficult to estimate this rule with few subjects” (Rubin and van der Laan, 2012). The bootstrap test (and our approach at large) looks at the holistic picture of the personalization scheme without focus on individual covariate-treatment interaction effects to determine if the personalization scheme in totality is useful, conceptually akin to the omnibus F-test in an OLS regression.

#### 3.5.1 Concerns With Using the Bootstrap for This Inference

There is some concern in the personalized medicine literature about the use of the bootstrap to provide inference. First, the estimator for *V* is a non-smooth functional of the data which may result in an inconsistent bootstrap estimator (Shao, 1994). The non-smoothness is due to the indicator function in Eq. 8 being non-differentiable, similar to the example found in (Horowitz, 2001, Chapter 52, Section 4.3.1). However, “the value of a fixed [response model] (i.e., one that is not data-driven) does not suffer from these issues and has been addressed by numerous authors” (Chakraborty and Murphy, 2014). Since our $\hat{V}$ is constructed out-of-sample, it is merely a difference of sample averages of the hold-out response values that are considered pre-sorted according to a fixed rule.^2^ This setup does not come without a substantial cost. Estimation of the improvement score out-of-sample means the effective sample size of our estimate is small and our power commensurately suffers. One can also implement the double bootstrap (see e.g. the comparisons in Chakraborty et al., 2010) herein and that is forthcoming in our software (see Section 5).

There is an additional concern. Some bootstrap samples produce null sets for the “lucky subjects” (i.e. P∪S=∅ of Table 2 or equivalently, all values of the indicator in Eq. 8 are zero). These are safe to ignore as we are only interested in the distribution of estimates conditional on feasibility of estimation. Empirically, we have noticed that as long as n>20, there are less than 1% of bootstrap samples that exhibit this behavior. Either way, we print out this percentage when using the PTE package and large percentages warn the user that inference is suspect.

### 3.6 Future Subjects

The implementation of this procedure for future patients is straightforward. Using the RCT data, estimate *f* to arrive at $\hat{f}$. When a new individual, whose covariates are denoted x_*_, enters a clinic, our estimated decision rule is calculated by predicting the response under both treatments, then allocating the treatment which corresponds to the better outcome, i.e. $\hat{d}$(x_*_). This final step is graphically illustrated in the top left of Figure 1.

It is important to note that $\hat{d}$(x_*_) is built with RCT data where treatment was allocated randomly and without regard to the subject covariates. In the example of the first order linear model with treatment interactions, the γ parameters have a causal interpretation—conditional causation based on the values of the moderating covariates. Thus $\hat{d}$(x_*_) reflects a treatment allocation that causes the response to be higher (or lower). We reiterate that this would not be possible with observational data which would suffer from elaborate confounding relationships between the treatment and subject covariates (see discussion in Sections 2 and 6.1).

## 4 Data Examples

We present two simulations in Sections 4.1 and 4.2 that serve only as illustrations that our methodology both works as purported but degrades in the case of pertinent information that goes missing. We then demonstrate a real clinical setting in Section 4.3.

### 4.1 Simulation With Correct Regression Model

Consider a simulated RCT dataset with one covariate *x* where the true response function is known:

$$Y=\beta_{0}+\beta_{1}X+A\left(\gamma_{0}+\gamma_{1}X\right)+ɛ,$$ (11)

where ɛ is mean-centered. We employ f(x,A) as the true response function, E[Y|X,A]. Thus, $d=d^{\text{*}}$, the “optimal” rule in the sense that a practitioner can make optimal allocation decisions (modulo noise) using $d\left(x\right)=1_{\gamma_{0}+\gamma_{1}x>0}$. Consider $d_{0}$ to be the **random** allocation procedure (see Section 3.3). Note that within the improvement score definition (Eq. 1), the notation $E_{X}^{d}\left[Y\right]$ is an expectation over the noise ℰ and the joint distribution of X,A. After taking the expectation over noise, the improvement under the model of Eq. 11 becomes

$$\begin{array}{l} \mu_{I_{0}}=E_{X}\left[\beta_{0}+\beta_{1}X+1_{\gamma_{0}+\gamma_{1}X>0}\left(\gamma_{0}+\gamma_{1}X\right)\right]-E_{X}\left[\beta_{0}+\beta_{1}X+0.5\left(\gamma_{0}+\gamma_{1}X\right)\right]\\ =E_{X}\left[\left(1_{\gamma_{0}+\gamma_{1}x>0}-0.5\right)\left(\gamma_{0}+\gamma_{1}X\right)\right]\\ =\gamma_{0}\left(\mathbb{P}\left(\gamma_{0}+\gamma_{1}X>0\right)-0.5\right)+\gamma_{1}\left(E_{X}\left[X1_{\gamma_{0}+\gamma_{1}x>0}\right]-0.5E_{X}\left[X\right]\right). \end{array}$$

We further assume $\mathrm{X∼}N\left(\mu_{X},\;\sigma_{X}^{2}\right)$ and we arrive at

$$\mu_{I_{0}}=\left(\gamma_{0}+\gamma_{1}\mu_{X}\right)\left(0.5-\Phi \left(-\frac{\gamma_{0}}{\gamma_{1}}\right)\right)+\gamma_{1}\frac{\sigma_{X}}{\sqrt{2\pi }}\exp\left(-\frac{1}{2\sigma_{X}^{2}}{\left(-\frac{\gamma_{0}}{\gamma_{1}}-\mu_{X}\right)}^{2}\right).$$

**FIGURE 2:**
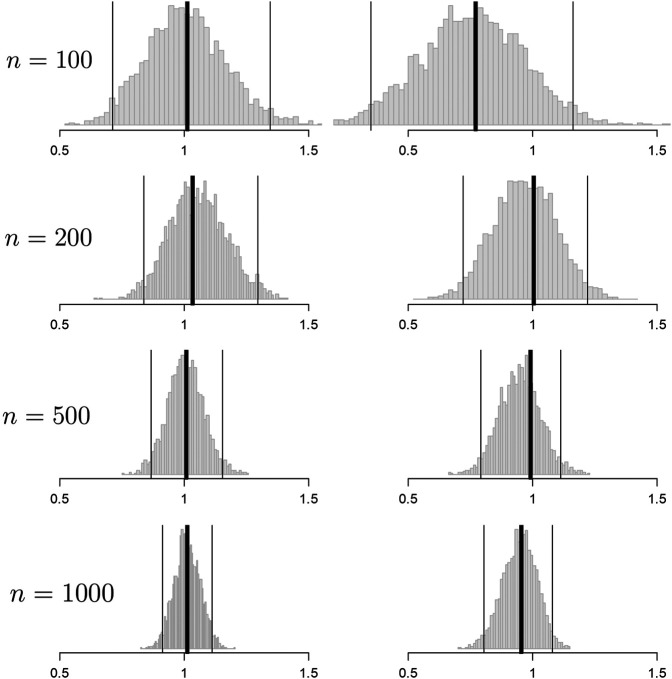
Histograms of the bootstrap samples of the out-of-sample improvement measures for $d_{0}$ **random** (left column) and $d_{0}$ **best** (right column) for the response model of Eq. 11 for different values of *n*. ${\hat{I}}_{0}$ is illustrated with a thick black line. The ${\text{CI}}_{\mu_{I_{0}},95\%}$ computed by the percentile method is illustrated by thin black lines.

We simulate under a simple scenario to clearly highlight features of our methodology. If $\mu_{X}=0,\sigma_{X}^{2}=1$ and $\gamma_{0}=0$, neither treatment $T_{1}$ or $T_{2}$ is on average better. However, if x>0, then treatment $T_{2}$ is better in expectation by $\gamma_{1}\times x$ and analogously if x<0, $T_{1}$ is better by $-\gamma_{1}\times x$. We then set $\gamma_{1}=\sqrt{2\pi }$ to arrive at the round number $\mu_{I_{0}}=1$. We set $\beta_{0}=1$ and $\beta_{1}=-1$ and let ${ℰ}_{i}{∼}^{iid}N\left(0,1\right)$. We let the treatment allocation vector *A* be a random block permutation of size *n*, balanced between $T_{1}$ and $T_{2}$. Since there is no PATE, the random and best $d_{0}$ procedures (see Section 3.3) are the same in value. We then vary n∈{100,200,500,1000} to assess convergence for both $d_{0}$ procedures and display the results in Figure 2.

Convergence to $\mu_{I_{0}}=1$ is observed clearly for both procedures but convergence for $d_{0}$ best is slower than $d_{0}$ rand. This is due to the $\hat{V}$ being computed with fewer samples: ${\bar{y}}_{\text{test}}$, which uses all of the available data, vs. ${\bar{y}}_{P\cup Q}$ or ${\bar{y}}_{R\cup S}$, which uses only half the available data on average (see Eqs. 6, 7). Also note that upon visual inspection, our bootstrap distributions seem to be normal. Our intuition is that non-normality in this distribution when using the software package warns the user that the inference is suspect.

In this section we assumed knowledge of *f* and thereby had access to an optimal rule. In the next section we explore convergence when we do not know *f* but pick an approximate model yielding a non-optimal rule that would still provide clinical utility.

### 4.2 Simulation With an Approximate Regression Model

Consider RCT data with a continuous endpoint where the true response model is

$$Y=\beta_{0}+\beta_{1}X+\beta_{2}U+A\left(\gamma_{0}+\gamma_{1}X^{3}+\gamma_{2}U\right)+ɛ,$$ (12)

where *X* denotes a covariate recorded in the RCT and *U* denotes a covariate that is not included in the RCT dataset. The optimal allocation rule $d^{\text{*}}$ is one when $\gamma_{0}+\gamma_{1}X^{3}+\gamma_{2}U>0$ and 0 otherwise. The practitioner, however, does not have access to the information contained in *U*, the unobserved covariate, and has no way to ascertain the exact relationship between *X* and the treatment. Consider the default model that is an approximation of the true population response surface,

$$f\left(X,A\right)=\beta_{0}+\beta_{1}X+A\left(\gamma_{0}+\gamma_{1}X\right),$$ (13)

which is different from the true response model due to (a) the misspecification of *X* (linear instead of cubic) and (b) the absence of covariate *U* (see Eq. 3). This is a realistic scenario; even with infinite data, $d^{\text{*}}$ cannot be located because of both ignorance of the true model form and unmeasured subject characteristics.

To simulate, we set the *X*’s, *U*’s and ℰ’s to be standard normal variables and then set $\beta_{0}=1,\beta_{1}=-1,\beta_{2}=0.5,\gamma_{0}=0,\gamma_{1}=1$ and $\gamma_{2}=-3$. The $X_{i}$’s and the $U_{i}$’s are deliberately made independent of one another so that the observed covariates cannot compensate for the unobserved covariates, making the comparison between the improvement under $d^{\text{*}}$ and *d* more stark. To find the improvement when the true model’s $d^{\text{*}}$ is used to allocate, we simulate under Eq. 12 and obtain $\mu_{I_{0}}^{\text{*}}\approx 1.65$ and analogously, to find the improvement under the approximation model’s *d*, we simulate under Eq. 13 and obtain $\mu_{I_{0}}\approx 0.79$. Further simulation shows that not observing *U* is responsible for 85% of this observed drop in improvement performance and employing the linear *X* in place of the non-linear $X^{3}$ is responsible for the remaining 15%. Since $\gamma_{0}=0$ and the seen and unseen covariates are mean-centered, there is no PATE and thus these simulated improvements apply to both the cases where $d_{0}$ is **random** and $d_{0}$ is **best**.

**FIGURE 3:**
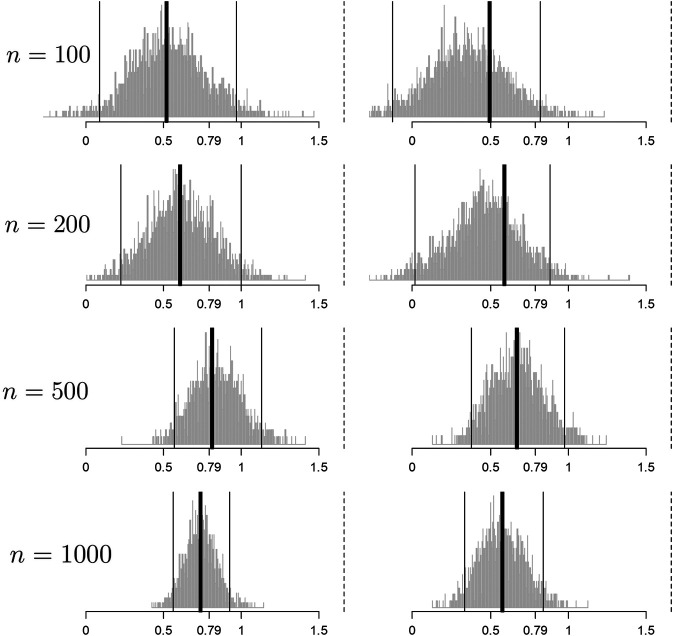
Histograms of the bootstrap samples of the cross-validated improvement measures for $d_{0}$ **random** (left column) and $d_{0}$ **best** (right column) for the response model of Eq. 12 for different values of *n*. ${\hat{I}}_{0}$ is illustrated with a thick black line. The ${\text{CI}}_{\mu_{I_{0}},95\%}$ computed via the percentile method is illustrated by thin black lines. The true population improvement $\mu_{I_{0}}^{\text{*}}$ given the optimal rule $d^{\text{*}}$ is illustrated with a dotted black line.

Figure 3 demonstrates results for n={100,200,500,1000} analogous to Figure 2. We observe that the bootstrap confidence intervals contain $\mu_{I_{0}}$ but not $\mu_{I_{0}}^{\text{*}}$. This is expected; we are not allocating using an estimate of $d^{\text{*}}$, only an estimate of *d*.

Convergence toward $\mu_{I_{0}}=0.79$ is observed clearly for both procedures and once again the convergence is slower for the best procedure for the same reasons outlined in Section 4.1. Note that the coverage illustrated here is far from $\mu_{I_{0}}^{\text{*}}$, the improvement using the optimal allocation rule. Kallus (2017) presents a coefficient of personalization metric similar to $R^{2}$ where a value of 100% represents perfect personalization and 0% represents standard of care. Here, we would fall far short of the 100%.

The point of this section is to illustrate what happens in the real world: the response model is unknown and important measurements are missing and thus any personalized medicine model falls far short of optimal. However, the effort can still yield an improvement that can be clinically significant and useful in practice.

There are many cases where our procedure will not find signal yielding improvement in patient outcomes either because it does not exist or we are underpowered to detect it. For example 1) in cases where there are many variables that are important and a small sample size. Clinical trials are not usually powered to find even single interaction effects, let alone many. The small sample size diminishes power to find the effect, similar to any statistical test. 2) If the true heterogeneity in the functional response cannot be approximated by a linear function. For instance, parabolic or sine functions cannot be represented whatsoever by best fit lines.

In the next section, we use our procedure in RCT data from a real clinical trial where both these limitations apply. The strategy is to approximate the response function using a reasonable model *f* built from domain knowledge and the variables at hand and hope to find demonstrate a positive, clinically meaningful $\mu_{I_{0}}$ knowing full well it will be much smaller than $\mu_{I_{0}}^{\text{*}}$.

### 4.3 Clinical Trial Demonstration

We consider an illustrative example in psychiatry, a field where personalization, called “*precision psychiatry*, promises to be even more transformative than in other fields of medicine” (Fernandes et al., 2017). However, evaluation of predictive models for precision psychiatry has been exceedingly rare, with a recent systematic review identifying 584 studies in which prediction models had been developed, with only 10.4% and 4.6% having conducted proper internal and external validation, respectively (Salazar de Pablo et al., 2020).

We will demonstrate our method (that provides this proper validation) on the randomized comparative trial data of DeRubeis et al. (2005). In this depression study, there were two treatments with very different purported mechanisms of action: cognitive behavioral therapy ($T_{1}$) and paroxetine, an antidepressant medication ($T_{2}$). After omitting patients who dropped out there were n=154 subjects with 28 baseline characteristics measured. Although this dataset did not have explicit patient-level identifying information, using these 28 characteristics could potentially identify some of the patients. Note that this study was funded and begun before clinical trials registration and thus it does not have a clinical trial registration number.

The primary outcome measure *y* is the continuous Hamilton Rating Scale for Depression (HRSD), a composite score of depression symptoms where lower means less depressed, assessed by a clinician after 16 weeks of treatment. A simple *t* test revealed that there was no statistically significant HRSD difference between the cognitive behavioral therapy and paroxetine arms, a well-supported finding in the depression literature. Despite the seeming lack of a population mean difference among the two treatments, practitioner intuition and a host of studies suggest that the covariates collected can be used to build a principled personalized model with a significant negative $\mu_{I_{0}}$. The lack of a difference also suggests that the **random** $d_{0}$ is an appropriate baseline comparison. If there were to be a clinically and statistically significant average difference in treatment outcomes between $T_{1}$ and $T_{2}$, then the **best** $d_{0}$ would be appropriate. In this latter case, we have found in our experience (in analyses of other datasets) that proving a personalization improvement is elusive as it is difficult to beat **best**. Even though the **best** $d_{0}$ is inappropriate in our context, we still provide its results in this section for illustrative purposes.

We now must specify a model, *f*. For the purpose of illustration, we employ a linear model with first-order interactions with the treatment (as in Eq. 4). Which of the 28 variables should be included in the model? Clinical experience and theory should suggest both prognostic (main effect) and moderator (treatment interaction) variables (Cohen and DeRubeis, 2018). We should not use variables selected using methods performed on this RCT data such as the variables found in DeRubeis et al. (2014), Table 3. Such a procedure would constitute data snooping and it will invalidate the inference provided by our method. The degree of invalidation is not currently known and is much needed to be researched.

**TABLE 3:** Baseline characteristics of the subjects in the clinical trial example for the moderating variables employed in our personalization model. These statistics differ slightly from those found in the table of DeRubeis et al. (2005, page 412) as here they are tabulated for subjects only after dropout (n=154).

Variable	Sample Average or Proportion
Age	40.3 ± 11.3
Chronicity	55.1%
Life stressors	6.6 ± 4.8
Personality disorder	48.1%
Unemployed	14.9%
Married	37.6%

Of the characteristics measured in this RCT data, previous researchers have found significant treatment moderation in age and chronicity (Cuijpers et al., 2012), early life trauma (Nemeroff et al., 2003) (which we approximate using a life stressor metric), presence of personality disorder (Bagby et al., 2008), employment status and marital status (Fournier et al., 2009) but almost remarkably baseline severity of the depression does not moderate (Weitz et al., 2015) and baseline severity is frequently the most important covariate in response models (e.g. Kapelner and Krieger, 2020, Figure 1B). We include these p=6 variables as moderators and as main effects^3^ and statistics of their baseline characteristics are found in Table 3.

**FIGURE 4:**
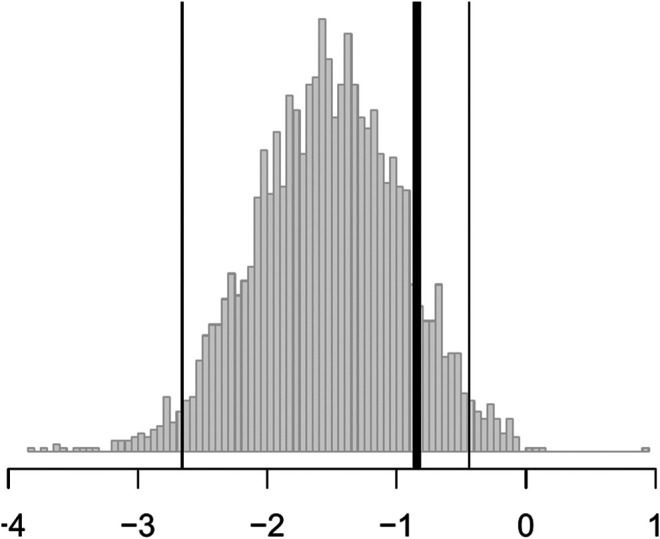
Histograms of the bootstrap samples of ${\tilde{I}}_{\text{Rand}}$ i.e. for the random$d_{0}$ business-as-usual allocation procedure. The thick black line is the best estimate of ${\hat{I}}_{0}$, the thin black lines are the confidence interval computed via the percentile method. More negative values are “better” as improvement is defined as lowering the HSRD composite score corresponding to a patient being less depressed.

The output from 3,000 bootstrap samples are shown in Figure 4. From these results, we anticipate that a new subject allocated using our personalization model will be less depressed on average by 0.84 HRSD units with a 95% confidence interval of [0.441, 2.657] compared to that same subject being allocated randomly to cognitive behavioral therapy or paroxetine. We can easily reject the null hypothesis that personalized allocation over **random** is no better for the new subject (*p* value = 0.001).

In short, the results are statistically significant, but the estimated improvement may not be clinically significant. According to the criterion set out by the National Institute for Health and Care Excellence, three points on the HRSD is considered clinically important. Nevertheless, this personalization scheme can be implemented in practice with new patients for a modest improvement in patient outcome at little cost.

## 5 The PTE Package

### 5.1 Estimation and Inference for Continuous Outcomes

The package comes with two example datasets. The first is the continuous data example. Below we load the library and the data whose required form is 1) a vector of length *n* for the responses, y and 2) a matrix X of dimension n×(p+1) where one column is named “treatment” and the other *p* are appropriate names of the covariates.


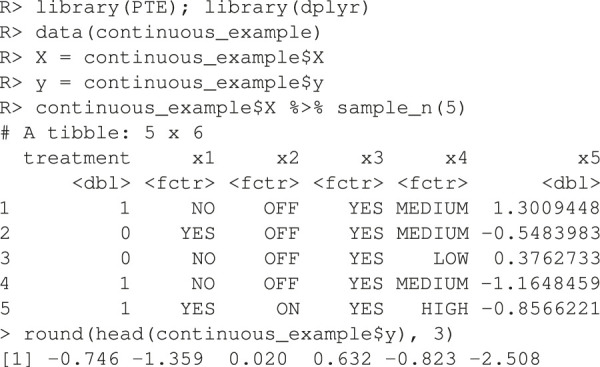


The endpoint ***y*** is continuous and the RCT data has a binary treatment vector appropriately named (this is required) and five covariates, four of which are factors and one is continuous. We can run the estimation for the improvement score detailed in Section 3.4.1 and the inference of Section 3.5 by running the following code:

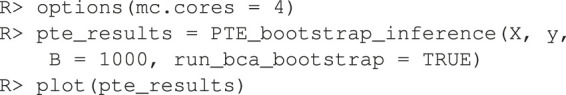

Here, 1,000 bootstrap samples were run on four cores in parallel to minimize runtime. The f model defaults to a linear model where all variables included are interacted with the treatment and fit with least squares. Below are the results.


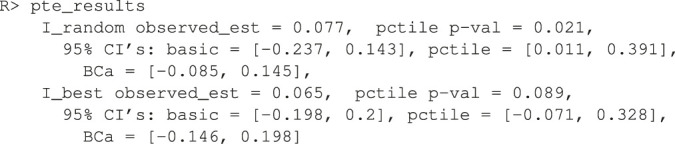


Note how the three bootstrap methods are different from another. The percentile method barely includes the actual observed statistic for the random comparison (see discussion in Section 3.5). The software also plots the $\tilde{I}$’s in a histogram (unshown).

To demonstrate the flexibility of the software, consider the case where the user wishes to use $x_{1},x_{2},x_{3},x_{4}$ as main effects and $x_{5}$ as the sole treatment moderator. And further, the user wishes to estimate the model parameters using the ridge penalty instead of OLS. Note that this is an elaborate model that would be difficult to justify in practice and it is only shown here as an illustration of the customizability of the software. Below is the code used to test this approach to personalization.


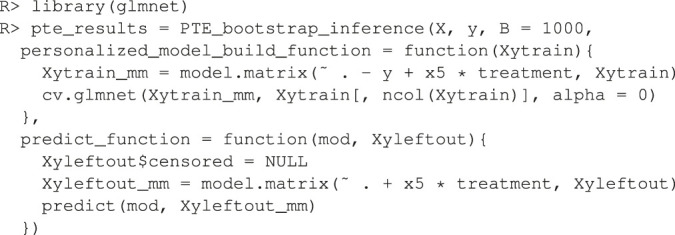


Here, the user passes in a custom function that builds the ridge model to the argument personalized_model_build_function. The specification for ridge employed here uses the package glmnet (Friedman et al., 2010) that picks the optimal ridge penalty hyperparameter automatically. Unfortunately, there is added complexity: the glmnet package does not accept formula objects and thus model matrices are generated both upon model construction and during prediction. This is the reason why a custom function is also passed in via the argument predict_function which wraps the default glmnet predict function by passing in the model matrix.

### 5.2 Estimation and Inference for Binary Outcomes

In order to demonstrate our software for the incidence outcome, we use the previous data but threshold its response arbitrarily at its 75th percentile to create a mock binary response (for illustration purposes only).


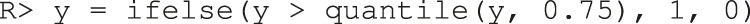


We then fit a linear logistic model using all variables as fixed effects and interaction effects with the treatment. As discussed in Section 3.4.2, there are three improvement metrics for incidence outcomes. The default is the odds ratio. The following code fits the model and performs the inference.


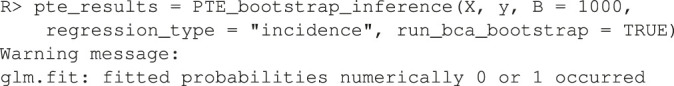


Note that the response type incidence has to be explicitly made known otherwise the default would be to assume the endpoint is continuous and perform regression. Below are the results.


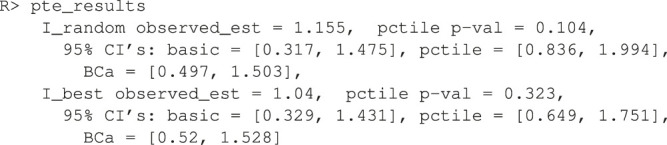


The *p* value is automatically calculated for $H_{0}:\mu_{I_{0}}<1$ (i.e. the odds of improvement is better in $d_{0}$ than *d*). Other tests can be specified by changing the H_0_mu_equals argument. Here, the test failed to reject $H_{0}$. Information is lost when a continuous metric is coerced to be binary. If the user wished to define improvement via the risk ratio (or straight probability difference), an argument would be added to the above, incidence_metric = “risk_ratio” (or “probability_difference”).

### 5.3 Estimation and Inference for Survival Outcomes

Our package also comes with a mock RCT dataset with a survival outcome. In addition to the required input data y, X described in Section 5.1, we now also require a binary vector c also of length *n* where a value $c_{i}=1$ denotes that the *i*th subject’s $y_{i}$ is a censored value. Below, we load the data.


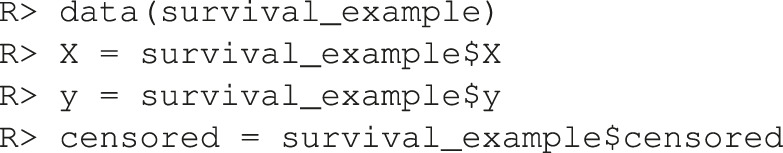


There are four covariates, one factor and three continuous. We can run the estimation for the improvement score detailed in Section 3.4.3 and inference for the true improvement by running the following code.


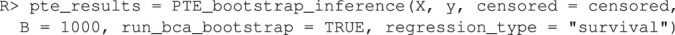


The syntax is the same as the above two examples except here we pass in the binary c vector separately and declare that the endpoint type is survival. Again by default all covariates are included as main effects and interactions with the treatment in a linear Weibull model.

In the default implementation for the survival outcome, improvement is defined as median survival difference of personalization vs. standard of care. The median difference can be changed via the user passing in a new function with the difference_function argument. The median difference results are below.


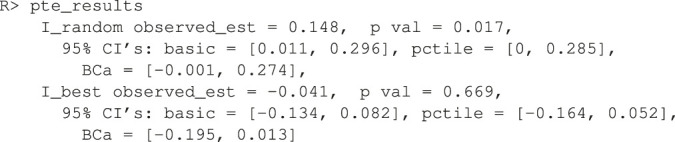


It seems that the personalized medicine model increases median survival by 0.148 vs. $d_{0}$ being the **random** allocation of the two treatments. If survival was measured in years (the typical unit), this would be about 2 months. However, it cannot beat the $d_{0}$ being the **best** of the two treatments. Remember, this is a much more difficult improvement metric to estimate as we are really comparing two cells in Table 2 to another two cells, one of which is shared. Thus the sample size is low and power suffers. This difficulty is further compounded in the survival case because censored observations add little information.

## 6 Discussion

We have provided a methodology to test the effectiveness of personalized medicine models. Our approach combines RCT data with a statistical model *f* of the response for estimating improved outcomes under different treatment allocation protocols. Using the non-parametric bootstrap and cross-validation, we are able to provide confidence bounds for the improvement and hypothesis tests for whether the personalization performs better compared to a business-as-usual procedure. We demonstrate the method’s performance on simulated data and on data from a clinical trial on depression. We also present our statistical methods in an open source software package in R named PTE which is available on CRAN. Our package can be used to evaluate personalization models generally e.g. in heart disease, cancer, etc. and even outside of medicine e.g. in Economics and Sociology.

### 6.1 Limitations and Future Directions

Our method and corresponding software have been developed for a particular kind of RCT design. The RCT must have two arms and one endpoint (continuous, incidence or survival). An extension to more than two treatment arms is trivial as Eq. 2 is already defined generally. Implementing extensions to longitudinal or panel data are simple within the scope described herein. And extending the methodology to count endpoints would also be simple.

Although we agree that a “once and for all” treatment strategy [may be] suboptimal due to its inflexibility” (Zhao et al., 2015), this one-stage treatment situation is still common in the literature and the real world and this is the setting we chose to research. We consider an extended implementation for dynamic treatment regimes on multi-stage experiments fruitful future work. Consider being provided with RCT data from sequential multiple assignment randomized trials (“SMARTs,” Murphy, 2005b) and an a priori response model *f*. The estimate of $\hat{V}\left[\hat{d}\right]$ (Eq. 5) can be updated for a SMART with *k* stages (Chakraborty and Murphy, 2014) where our Table 2 is a summary for only a single stage. In a SMART with *k* stages, the matrix becomes a hypercube of dimension *k*. Thus, the average of diagonal entries in the multi-dimensional matrix is the generalization of the estimate of $\hat{V}\left[\hat{d}\right]$ found in Eq. 6. Many of the models for dynamic treatment regimes found in Chakraborty and Moodie (2013) can then be incorporated into our methodology as *d*, and we may be able to provide many of these models with valid statistical inference. Other statistics computed from this multi-dimensional matrix may be generalized as well.

Our choices of $d_{0}$ explored herein were limited to the **random** or the **best** procedures (see Section 3.3). There may be other business-as-usual allocation procedures to use here that make for more realistic baseline comparisons. For instance, one can modify **best** to only use the better treatment if a two-sample *t*-test rejects at prespecified Type I error level and otherwise default to **random**. One can further set $d_{0}$ to be a regression model or a physician’s decision tree model and then use our framework to pit two personalized medicine models against each other.

It might also be useful to consider how to extend our methodology to observational data. The literature reviewed in Section 2 generally does not require RCT data but “only” a model that accurately captures selection into treatments e.g. if “the [electronic medical record] contained all the patient information used by a doctor to prescribe treatment up to the vagaries and idiosyncrasies of individual doctors or hospitals” (Kallus, 2017, Section 1). This may be a very demanding requirement in practice. In this paper, we do not even require valid estimates of the true population response surface. In an observational study one would need that selection model to be correct and/or a correct model of the way in which subjects and treatments were paired (Freedman and Berk, 2008). Although assuming one has a model that captures selection, it would be fairly straightforward to update the estimators of Section 3.4 to inverse weight by the probability of treatment condition (the “IPWE”) making inference possible for observational data (Zhang et al., 2012b; Chakraborty and Murphy, 2014; Kallus, 2017).

Another extension would be to drop the requirement of specifying the model *f* whose specification is a tremendous constraint in practice: what if the practitioner cannot construct a suitable *f* using domain knowledge and past research? It is tempting to use a machine learning model that will both specify the structure of *f* and provide parameter estimates within e.g. Kallus’s personalization forests (Kallus, 2017) or convolutional neural networks (LeCun and Bengio, 1998) if the raw subject-level data had images. We believe the bootstrap of Section 3.5 will withstand such a machination but are awaiting a rigorous proof. Is there a solution in the interim? As suggested as early as Cox (1975), we can always pre-split the data in two where the first piece can be used to specify *f* and the second piece can be injected into our procedure. The cost is less data for estimation and thus, less power available to prove that the personalization is effective.

If we do not split, all the data is to be used and there are three scenarios that pose different technical problems. Under one scenario, a researcher is able to specify a suite of possible models before looking at the data. The full suite can be viewed as comprising a single procedure for which nonparametric bootstrap procedures may in principle provide simultaneous confidence intervals (Buja and Rolke, 2014). Under the other two scenarios, models are developed inductively from the data. This problem is more acute for instance in Davies (2015) where high-dimensional genomic data is incorporated for personalization (e.g. where there are many more SNPs than patients in the RCT). If it is possible to specify exactly how the model search is undertaken (e.g., using the lasso), some forms of statistical inference may be feasible. This is currently an active research area; for instance, Lockhart et al. (2014) and Lee et al. (2016) develop a significance test for the lasso and there is even some evidence to suggest that the double-peeking is not as problematic as the community has assumed (Zhao et al., 2020).

Our method’s generalizability to future patients is also in question as our validation was done within the patients of a RCT. The population of future patients is likely not the same as the population of patients in the RCT. Future patients will likely have wider distributions of the *p* covariates as typical RCTs feature strict inclusion criteria sometimes targeting high risk patients for higher outcome event rates. A good discussion of these issues is found in Rosenberger and Lachin (2016), Chapter 6. The practitioner will have to draw on experience and employ their best judgment to decide if the estimates our methodology provides will generalize.

And of course, the method herein only evaluates if a personalization scheme works on average over an entire population. “Personalized medicine” eponymously refers to personalization for an individual. Ironically, that is not the goal herein, but we do acknowledge that estimates and inference at an individual level coupled to valid inference for the improvement score is much-needed. This is not without difficulty as clinical trials are typically not powered to examine subgroup effects. A particularly alarming observation is made by Cuijpers et al. (2012), page 7, “if we want to have sufficient statistical power to find clinically relevant differential effect sizes of 0.5, we would need …. about 23,000 patients”.

## Data Availability

The datasets presented in this article in Section 4.3 is not readily available because the clinical data cannot be anonymized sufficiently according to the IRB guidelines of the institutions where the study was performed. For more information, contact the authors of the study.

## Footnotes

1 Note that this problem is encountered in fields outside of just medicine. For example, finding the movie that will elicit the most enjoyment to the individual (Zhou et al., 2008) or assessing whether a certain unemployed individual can benefit from job training (LaLonde, 1986). Although the methods discussed herein can be applied more generally, we will employ examples and the vocabulary from the medical field.

2 Note also that we do not have the additional non-smoothness created by Q-learning during the maximization step (Chakraborty et al., 2010, Section 2.4).

3 Although this is standard linear modeling practice, it is not absolutely essential in our methodology, where our goal is neither inference for the variables nor prediction of the endpoint.
